# Prevalence and determinants of HPV infection among Colombian women with normal cytology

**DOI:** 10.1038/sj.bjc.6600442

**Published:** 2002-08-01

**Authors:** M Molano, H Posso, E Weiderpass, A J C van den Brule, M Ronderos, S Franceschi, C J L M Meijer, A Arslan, N Munoz, the HPV Study Group

**Affiliations:** Department of Pathology, Unit of Molecular Pathology, Vrije Universiteit Medical Centre, De Boelelaan 1117, 1081 HV, Amsterdam, The Netherlands; Division de Investigacion, Instituto Nacional de Cancerologia, Bogota, Colombia; Unit of Field and Intervention Studies, International Agency for Research on Cancer, Lyon, France

**Keywords:** HPV, Colombia, epidemiology, cervix uteri

## Abstract

Human papillomavirus is the principal risk factor associated with cervical cancer, the most common malignancy among women in Colombia. We conducted a survey, aiming to report type specific prevalence and determinants of human papillomavirus infection in women with normal cytology. A total of 1859 women from Bogota, Colombia were interviewed and tested for human papillomavirus using a general primer GP5+/GP6+ mediated PCR–EIA. The overall HPV DNA prevalence was 14.8%; 9% of the women were infected by high risk types, 3.1% by low risk types, 2.3% by both high risk/low risk types and 0.4% by uncharacterized types (human papillomavirus X). Thirty-two different human papillomavirus types were detected, being human papillomavirus 16, 58, 56, 81(CP8304) and 18 the most common types. The human papillomavirus prevalence was 26.1% among women younger than 20 years, 2.3% in women aged 45–54 years, and 13.2% in women aged 55 years or more. For low risk types the highest peak of prevalence was observed in women aged 55 years or more. Compared to women aged 35–44 years, women aged less than 20 years had a 10-fold increased risk of having multiple infections. Besides age, there was a positive association between the risk of human papillomavirus infection and number of regular sexual partners and oral contraceptive use. In women aged below 25 years, high educational level and having had casual sexual partners predicted infection risk. In conclusion, there was a broad diversity of human papillomavirus infections with high risk types being the most common types detected. In this population multiplicity of sexual partners and, among young women, high educational level and casual sexual partners seem to determine risk.

*British Journal of Cancer* (2002) **87**, 324–333. doi:10.1038/sj.bjc.6600442 www.bjcancer.com

© 2002 Cancer Research UK

## 

Cervical cancer is the most common cancer among women in developing countries, and the sixth most common cancer in women in developed countries (Parkin *et al*, 1999). In Colombia, where the annual standardised incidence rate of cancer of the cervix is high (23 per 100 000), it is also the principal cause of cancer mortality among women (Globocan, 1998).

Infection with certain types of human papillomavirus (HPV) is the main cause of cervical cancer and its precursor lesions (van Den Brule *et al*, 1991; Muñoz *et al*, 1992; Chichareon *et al*, 1998; Ngelangel *et al*, 1998; Rolon *et al*, 2000). HPV infection is now considered as virtually necessary but not sufficient to cause this malignancy (Walboomers *et al*, 1999). Other host factors such as genetic alterations, endogenous female hormones and immune status or environmental factors (e.g. smoking and use of exogenous hormones) are also likely to be involved in cervical carcinogenesis (Bosch *et al*, 1992; Ngelangel *et al*, 1998; Gostout *et al*, 1998; Palefsky *et al*, 1999).

On the basis of their main association with benign or (pre-) malignant lesions, HPVs can be subdivided into low risk (LR) and high-risk (HR) oncogenic types. Women with normal cervical cytology who are infected with HR HPV have an approximately 100-fold increased risk of developing CIN III compared to uninfected women (Rozendaal *et al*, 1996). Therefore, it has been suggested that HR HPV detection might be used as a tool to identify women at high risk of cervical cancer, in addition to – or as an alternative to – Pap smears (Rozendaal *et al*, 1996, 2000; Meijer *et al*, 1998).

The marked geographic differences in incidence of cervical cancer are not only the result of differences in screening patterns, but also of differences in exposure to risk factors. For example, the very low incidence rates (2–4 per 100 000) reported from some areas in China and Spain (Parkin *et al*, 1997) where population-based screening programs do not exist, are probably the result of low exposure to HPV resulting from the conservative sexual behaviour prevalent in the previous decades.

To investigate geographic differences in the prevalence of type-specific HPV infection and of other risk factors for cervical cancer, the International Agency for Research on Cancer is co-ordinating a series of surveys of prevalence of HPV infection and CIN lesions in countries with high and low incidence of cervical cancer, this study being one of them.

The prevalence of HPV, as detected by HPV DNA, characteristically peaks after initiation of sexual activity and decreases with age. A second peak in peri-menopausal women has been recently described in some populations (Cuzick *et al*, 1999; Herrero *et al*, 2000; Lazcano *et al*, 2001). It is currently unknown whether different age-specific curves of HPV prevalence are attributable to an effect of age *per se* or to differences in risk between birth cohorts.

A prospective cohort study in Bogota, Colombia, was initiated in the early 1990s by the Colombian National Institute of Cancer and the International Agency for Research on Cancer (IARC). The aim of this study was to investigate the natural history of HPV infection and CIN lesions among low-income Colombian women. We report here the results of a cross-sectional analysis of the cohort at enrolment, with focus on determinants of HPV infection among women with normal cervical cytology.

## MATERIALS AND METHODS

### Study population

Between November 1993 and November 1995 the Colombian National Cervical Cancer Control Program conducted a census in four health districts in Bogota, which had no previously implemented population-based cervical screening program. The first 2000 women aged 18–85 years identified in the census were invited to participate in the study. Additionally, 200 adolescents aged 13–17 years were invited to participate. They were identified consecutively from an adolescent clinic giving contraceptive counselling in the study area. At recruitment, all women were interviewed face-to-face by specially trained interviewers. They answered to a structured questionnaire on socio-demographic characteristics, lifelong sexual behaviour, reproductive and menstrual history, and smoking and dietary habits. After interview all women underwent a pelvic examination.

From the 2200 invited women, 53 refused to participate, eight were considered ineligible (mental illness, hysterectomy, history of cervical cancer) and 29 did not provide cell specimens for HPV DNA detection, so that 2110 women had cell specimens to be analysed. Of them 150 had abnormal cytology (according to the Bethesda system classification) and 101 of the women with normal cytology were beta-globin PCR negative (which is considered an indicator of poor DNA quality). After these exclusions, 1859 women remained in the analysis presented here. In the multivariate analysis (see below) we further excluded 14 women who did not answer the questionnaire completely, resulting in 1845 women with normal cytology and risk factors analysis. Informed consent was obtained from all participants included in the study. The local ethical committee and the ethical committee at IARC approved the study protocol.

### Biological specimens

During the gynaecological examination, cervical scrapes were collected from each woman using two Ayre spatulas and two endocervical brushes. The first spatula and brush were used for a routine Pap smear, which was classified according to the Bethesda system terminology. The second spatula and brush and the remaining cells of the first spatula and brush, were placed in a tube containing 5 ml of phosphate-buffered saline (PBS 1×)+0.05% thiomersal. Cells were detached from the spatula and endocervical brush through vortex, and subsequently centrifuged at 3000 **g** for 10 min. The cell pellet was resuspended in 1 ml buffer Tris-HCl 10 mM pH 8.3 and stored at −70°C until their use. For analysis, 100 μl aliquots were boiled for 10 min at 100°C, cooled on ice and centrifuged for 1 min at 3000×g. 10 μl of these pre-treated crude cell suspensions were used for PCR analysis (van den Brule *et al*, 1990; de Roda Husman *et al*, 1994). To assess the quality of the target DNA, all pellets were pre-screened using a 209 base pair amplifying β-globin PCR using the primer combination BPCO3 and BPCO5 as described by de Roda Husman *et al* (1995b).

### HPV detection by PCR

HPV-DNA detection was performed by a standard GP5+/GP6+ PCR based assay, as described by de Roda Husman *et al* (1995a). Briefly, the GP–PCR reaction was carried out using 50 μl of PCR solution containing 10 mM Tris HCL pH 8.3, 50 mM KCl, 200 μM of each deoxynucleotide, 3.5 mM of MgCl_2_, 1 U of DNA polymerase (AmpliTaq; Perkin-Elmer, USA) and 25 pmol of each of the GP5+ and biotinylated GP6+ primers (Eurogentec, Belgium): 40 cycles of amplification were carried out using a Perkin-Elmer 9600,USA thermocycler. Each cycle included a denaturation step at 95°C for 1 min, one annealing step at 40°C for 1 min, and a chain elongation step at 72°C for 1.5 min. The first step was preceded by a denaturation step of 4 min and the last step was followed by an elongation step of 10 min.

Three dilutions of the cell line SiHa containing 1–10 copies of HPV16 (100 pg, 1 ng and 10 ng) were used as positive control. As negative PCR controls, distilled water and processing blanks were used every 10 samples. HPV positivity was assessed by Southern blot hybridization of GP5+/GP6+ PCR products with a cocktail probe of specific [α-^32^P]dCTP labelled DNA fragments from cloned DNA of HPV6, 11, 16, 18, 31 and 33 under low stringent conditions (van den Brule *et al*, 1990; de Roda Husman *et al*, 1995a,b).

### Type specific HPV detection

HPV positive samples were first subjected to HPV group-specific analysis using cocktail probes for HR and LR HPVs (Jacobs *et al*, 1997, 2000). The HR HPV cocktail probe consisted of oligoprobes for HPV16, 18, 31, 33, 35, 39, 45, 51, 52, 56, 58, 59, 66 and 68 and the LR HPV consisted of oligoprobes for HPV 6, 11, 40, 42, 43, 44, HPV 82 (MM4), HPV83 (MM7), HPV84 (MM8), Iso39, HPV71 (CP8061), CP6108, HPV 81 (CP8304), HPV 26, 34, 53, 54, 55, 57, 61, 70, 72, 73. However HPV types with unknown oncogenic potential – namely 26, 53, 73, 34 and Iso 39 – were considered in the results as HR types, based on both the alignment analysis of the E6 gene (modified from Myers *et al*, 1996) and the risk estimates obtained for the various HPV types within a multi-center case–control study of cervical cancer conducted by the IARC (Muñoz *et al*, 1992, Muñoz, 2000; Bosch *et al*, 1995; Chaouki *et al*, 1998; Chichareon *et al*, 1998; Ngelangel *et al*, 1998; Rolon *et al*, 2000).

Briefly, in the enzyme immune assay (EIA), 5 μl of the biotinylated PCR products were captured in streptavidine-coated microtitle plates (Roche, Mannheim, Germany). Subsequently, the wells were washed three times with 1×SSC; the captured DNA was denatured by alkaline treatment with 0.1 M NaOH and hybridised to digoxigenin-labelled type-specific oligoprobes. After several washings the hybrids were detected using anti-digoxigenin (Fab fragments) labelled with alkaline phosphatase (Roche, Mannheim, Germany) and paranitrophenyl phosphate (Sigma, USA) was used as substrate. The optical density (OD) was measured at 405 nm using a Labsystem Multiscan reader. In our assay a cut-off point was defined using three times mean OD of the negative controls.

The HPV positive samples were analysed successively in other specific EIA probe sub-cocktails and finally specific oligoprobes were used to identify each individual HPV type. Samples which were positive by Southern blot analyses and negative by HR and LR EIA, were considered as positive for HPV of undetermined type (HPV X).

### Data analysis

We computed odds ratios (ORs) and 95% confidence intervals (CI) using unconditional logistic regression models, considering HPV infections as dependent variables and several known or hypothesised risk factors for cervical cancer as independent variables (STATA; Stata Press, College Station, TX, USA). We performed both age adjusted (grouped as <20, 20–24, 25–29, 30–34, 35–39, 40–44, 45–54, 55 or more years) and multivariate analyses. The following variables were included in the models: age (as categorised above), educational level (low, intermediate and high), number of regular (1, 2, 3 or more) sexual partners, parity (0, 1–2, 3 or more children), age at first sexual intercourse (less 17, 17–19, 20 or more years), use of oral contraceptives (ever or never use), condoms (ever or never use), and smoking habits. The different age-adjusted and multivariate models did not differ materially from each other. Therefore we present in this report only results of the fully adjusted multivariate models.

## RESULTS

### Characteristics of the study population

The characteristics of the study population are summarised in Table 1

**Table 1:**
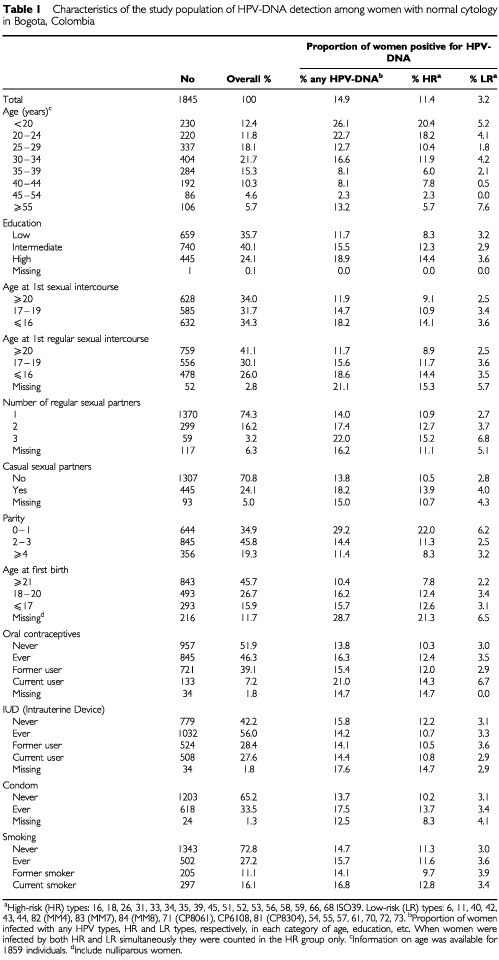
Characteristics of the study population of HPV-DNA detection among women with normal cytology in Bogota, Colombia

. Most of the participating women were aged 25–34 years (median age 32), had low (36%) or intermediate (40%) educational level, had their first sexual intercourse or first regular sexual partner before the age of 20 (median 18 and 19 years, respectively), reported a single regular lifelong sexual partner (74%), and had at least two full term pregnancies (median=2). Intrauterine device (IUD) was the most common contraceptive method ever used (56%), followed by oral contraceptives (46.3%) and condoms (33.5%). Only one third of the women had ever smoked regularly.

### HPV-DNA prevalence

The overall HPV-DNA prevalence rate was 14.9% (Table 1), and 32 different HPV types were detected. Of the HPV-DNA positive women, 9% were infected with HR types only, 3.2% with LR types only, and 0.4% with HPV X. 2.3% of women were infected with both HR/LR types, which were grouped together with HR types in all analysis. Thus the total HR type prevalence was 11.4% (Table 1). HPV-DNA age-specific prevalence was highest among women below age 20 (26%), and lowest among women aged 45–54 years (2.3%). Women aged 55 or more years had a prevalence rate of 13.2% (Table 1).

**Figure 1:**
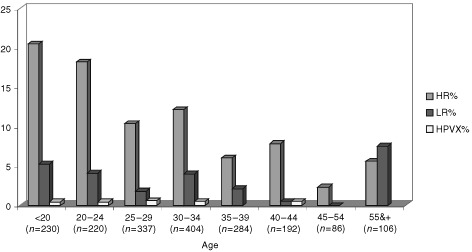
Prevalence of high and low risk of human papillomavirus among women with normal cytology from Bogota, Colombia

HR HPV types were at least three times more prevalent than LR types in all age groups, except among women aged 55 or more years (Table 1). Similarly to the overall HPV prevalence, the HR age-specific prevalence rate curve presented a U-shape (Figure 1

), the highest prevalence being among women aged less than 20 years (20.4%), the lowest among women aged 45–54 years (2.3%), and an intermediate among women aged 55 years or more (5.7%). For LR types, the age specific pattern was rather different: while no women aged 45–54 years were positive, those aged 55 years or more had the highest prevalence rate (7.6%) of all age groups. Thus, among older women, LR types were more common than HR types (Figure 1).

Single infections (infections with only one HPV type) were detected in 194 women (10.4% of the entire study population; 70.3% of the HPV positive women). Among these women, the most common HPV HR types were 16 (16.3% of the women with single infections), 58 (6.2%), 56 (3.6%) and 18 and 51 (2.9%). The most frequent HPV LR types were HPV 81 (CP8304) (3.6%), 42 (2.5%), 40 (1.9%) and 70 (1.4%). The prevalence of single HPV infection decreased with age, the same HPV types being the commonest ones in all age groups (Table 2

**Table 2:**
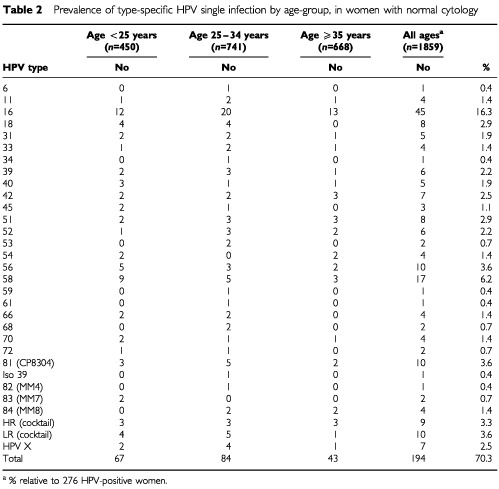
Prevalence of type-specific HPV single infection by age-group, in women with normal cytology

).

Multiple infections (infections with two or more HPV types) were detected in 82 women (4.4% of the entire study population; 29.7% of the HPV positives). Women aged less than 25 years had an almost five times higher prevalence rates of multiple infections (9.6%) than women aged 35 years or more (1.9%). Most of the multiple infections were caused by HR/LR types (53.7%) or HR/HR types (42.7%) and less (3.6%) by LR/LR types. Among women with multiple infections, HR types were present in 97.6% of those women aged less than 25 years, 100% among those aged 25–34 years, and 85.7% among those aged 35–54 years, and 83.3% among those aged 55 or more years. HPV 35, 43, 44 and CP6108 were detected only in multiple infections (data not shown).

### Risk factors for HPV infections

Besides age, number of regular sexual partners and OC use, no other risk factors were clearly associated with overall risk for HPV infections in our study population (Tables 1 and 3

**Table 3:**
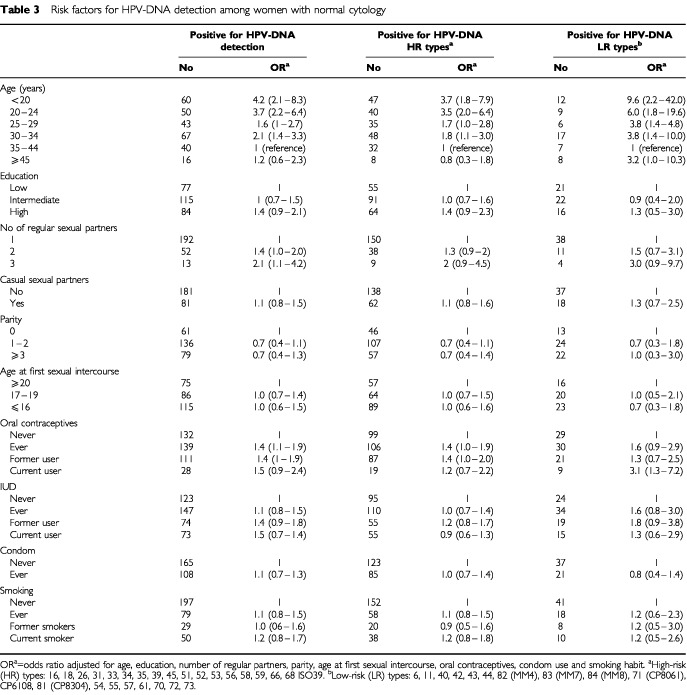
Risk factors for HPV-DNA detection among women with normal cytology

). For HR HPV infections, there was a trend of a decreased risk among women with parities (OR 0.7), and of an increased risk among users of oral contraceptives (OR 1.4, 95% CI 1.0–1.9). For LR types, current use of oral contraceptives increased the infection risk significantly (OR 3.1, 95% CI 1.3–7.2) and use of IUD also tended to increase the risk of infection (OR 1.6, 95% CI 0.8–3.0 – Table 3). Although there was no statistically significant interaction between the effects of age and other potential risk factors for HPV infections, some interesting age-specific patterns emerged in the analysis, of which some are presented in Table 4

**Table 4:**
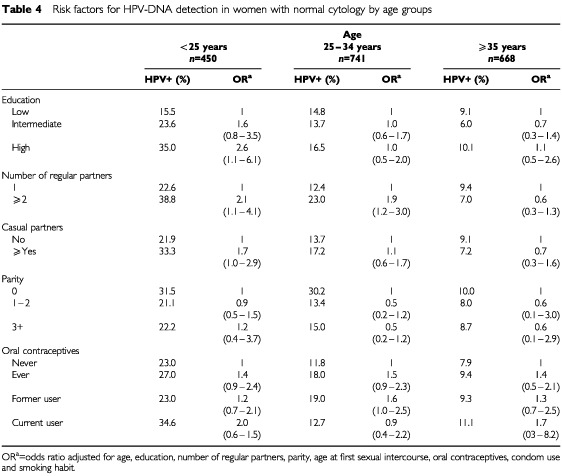
Risk factors for HPV-DNA detection in women with normal cytology by age groups

.

In women below the age of 25 years, high educational level (OR 2.6, 95% CI 1.1–6.1), and more than a single regular (OR 2.1, 95% CI 1.1–4.1) or casual (OR 1.7, 95% CI 1.0–2.9) sexual partner predicted infection risk (Table 4). There was also a suggestion of an increased risk with current use of oral contraceptives (OR 2.0, 95% CI 0.6–1.5 – Table 4). The addition of women attending an adolescent clinic (age 13–17 years) did not generate a bias in the study results, since no statistically significant differences were found when they were excluded from the analysis. The association with number of partners persisted with a slightly lower odds ratio (OR 1.7, 95% CI 0.7–4.2). On the other hand, the association with education was stronger (OR 7.4, 95% CI 1.9–29.0 for high level of education).

In the age group 25–34 years, having had more than one regular sexual partner increased HPV infection risk (OR 1.9, 95% CI 1.3–3.0 – Table 4). There was no clear association between the existence of casual sexual partners, age at first sexual intercourse or first birth, use of IUDs or condoms and risk. Former use of oral contraceptives increased the infection risk slightly (OR 1.6, 95% CI 1.0–2.5) and parity slightly decreases the risk (OR 0.5, 95% CI 0.2–1.2). No clear pattern of association was observed among women aged 35 or more years. The study had insufficient women of the age of 55 years or more to allow further subgroup analysis with meaningful statistical power.

Young age and high level of attained education were better predictors of multiple infections than of single ones (Table 5

**Table 5:**
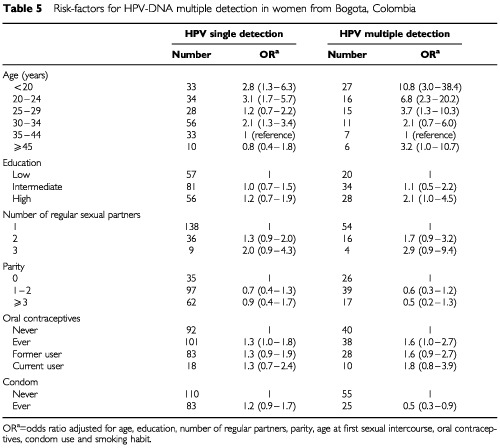
Risk-factors for HPV-DNA multiple detection in women from Bogota, Colombia

). For multiple infections, there was a trend for a protective effect of parity (for three or more children compared to nulliparous, OR 0.5, 95% CI 0.2–1.3) and use of condoms (OR 0.5, 95% CI 0.3–0.9) (Table 5). In most instances, however, examined characteristics shared similar associations with single and multiple infections.

## DISCUSSION

To date, few studies evaluated age-specific HPV prevalence patterns and determinants of HPV infection among women with normal cytology in countries with high incidence of cervical cancer. We report here the first investigation of this type done in Colombia. An overall HPV DNA prevalence of 14.9% was found, which is similar to that reported in other high-risk populations in Mexico (14.5%) (Lazcano *et al*, 2001) and Costa Rica (16%) (Herrero *et al*, 2000). In addition, it is similar to the HPV-DNA prevalence among control women (13%) in a concurrent case–control study in Cali, Colombia (Muñoz *et al*, 1996).

Several studies have suggested that the prevalence of HPV infection decreases with age, HPV being uncommon in cytological normal women over age 35 years (Melkert *et al*, 1993; Morrison, 1994). However, a few recent large population-based surveys from Costa Rica and Mexico also presented some increase in HPV infection among women in peri- and post-menopausal ages. While in Costa Rica the peak was observed in women older than 55 years, with a predominance of LR HPV types, in Mexico the second peak started earlier, after 45 years of age, with predominance of HR HPV types and with an increase in detection of LR HPV types compared to younger ages.

In our study the HPV prevalence in the age group 45–54 years was low, but there was an increased prevalence of HPV infection in women aged 55 or more years, with a predominance of LR types and multiple HPV infections, but still 5.7% were HR HPV positive. If confirmed, the risk of HPV infection – most notably LR types – among post-menopausal women, it may be explained in different ways: (1) reactivation of latent HPV infections by decreased immune response. Some immunological studies show a decrease in circulating mature T cells in older people as a result of a decrease in CD8+ lymphocytes, a decline in the frequency of CD4+ T cells producing IL-2 and/or a decreased expression in IL-2 receptors (Lesourd and Meaume, 1994; Gostout *et al*, 1998; Ginaldi *et al*, 1999). (2) Reactivation of latent HPV infections by hormonal changes related to the gradual decline of ovarian function around menopause (Lazcano *et al*, 2001). (3) A cohort effect (Herrero *et al*, 2000), where older women were exposed to the virus early in life and belonged to generations more heavily exposed to HPV infections.

An alternative explanation for this second peak lies in the fact that only women with normal cervical cytology were included in this study. Persistent infections with HR HPV types will lead to cervical intraepithelial lesions with subsequent treatment. It may result in an under-representation of HR HPV types and the proportion of them in the population will diminish at older age. In contrast, LR HPV infections that rarely give rise to cervical dysplasia will remain (Jacobs *et al*, 2000).

We identified 32 different HPV types in our study population. This broad diversity of infections is consistent with previous studies showing a greater HPV heterogeneity in mild cervical dysplasias than in severe dysplasias (de Roda Husman *et al*, 1994; Liaw *et al*, 1999; Jacobs *et al*, 2000; Lazcano *et al*, 2001). It has been estimated that at least 50% of sexually active adults have ever had a genital HPV infection, most of these, however, being transient and resolving spontaneously (Koutsky, 1997).

In our study HPV 16, 58, 56, 52, HPV 81 (CP8304), 51 and 18 were the most prevalent HPV types detected as single infections in all age groups. Although HPV16 is the predominant type detected in the majority of studies (Jacobs *et al*, 2000; Lazcano *et al*, 2001; Rolon *et al*, 2000), remarkable differences were noted in our study population with a high prevalence of other HPV types such as HPV 56, 58 and the LR type HPV 81 (CP8304). This information should be taken into account when developing HPV vaccines tailored to this population.

In this study HPV 26, 53, 73, 34 and Iso 39 were analysed as types with oncogenic potential according their presence in patients with cervical cancer and the alignment analysis of the E6 gene (modified from Myers *et al*, 1996). However the percentage of these HPVs is very low and for absolute HR or LR classification, transformation studies in combination with follow up epidemiological studies are necessary.

The presence of multiple infections (29.7%) was higher than previously observed among control subjects in the IARC studies done in Brazil (0%) (Eluf-Neto *et al*, 1994), the Philippines (14.3%) (Ngelangel *et al*, 1998) Thailand (9.8%) (Chichareon *et al*, 1998), Morocco (5.3%) (Chaouki *et al*, 1998) and Paraguay (16.7%) (Rolon *et al*, 2000), lower than in a population-based study from Costa Rica (39%) (Herrero *et al*, 2000) and similar to the results from a study done in The Netherlands (28%) (Jacobs *et al*, 2000), where 3305 cytological normal cervical scrapes were analysed using the same laboratory technique as in the present study. These differences in prevalence of multiple infections could be due to differences in the technique used (sensitivity, specificity and types identified), or real differences in the prevalence of the HPV types searched for and identified in the populations studied. In addition, these populations differed in age-composition: mean age 32 years in this study, whereas in Thailand it was 49.7 years. Taking into account the age-dependent prevalence of multiple infections, this fact may also explain the discrepancy in results. In our study, 97% of the women with multiple infections presented a HR HPV type. The long-term follow up of these infections will help to clarify the role of HR multiple infections in the development of cervical lesions.

Besides age, number of regular sexual partners and OC use (specially in women below age 35 years) which were risk factors for HPV positivity, other reproductive and sexual behaviour factors considered by us were only weakly and inconsistently related to HPV infection. It might be explained by the relatively high frequency of HPV infections in the studied women and possibly the predominance of ‘male role’ in the transmission of HPV infections to women (particularly over age 35 years). Unfortunately, no information was available on the sexual behaviour of the partners in our study population. Our results suggest an age-dependent association between HPV infection and some risk factors. A possible explication of the highest prevalence of HPV infections among young, highly educated women and with more than one regular or casual sexual partner could reflect changes in lifestyle and sexual behaviour in younger generations (possibly reduced influence of religion and greater freedom).

We observed an increase of HPV infections and particularly of LR types in women older than 55 years and we tried to analyse if these women were different in terms of exposure to risk factors. Due to the low number of women in this group we could not find a clear pattern of association. We found that use of oral contraceptives may be a risk factor for both HR and LR HPV infections. Use of exogenous hormones – as contraceptives or hormone replacement – could influence HPV infections in different ways. Results from laboratory work have indicated that the HPV genome contains a hormone-recognition segment, which might show an interactive effect of oral contraceptives and HPV (Monsonego *et al*, 1991). Additionally, steroid hormones do interfere with cellular gene function involved in cell cycle regulation and apoptosis, and they also might inhibit the immunologically mediated resolution of minor HPV-induced cervical lesions (Von Knebel Doeboritz *et al*, 1997).

With respect to parity, in our study there was a trend of decreased risk of HR and multiple HPV infections with increase of number of births. Previous studies have presented conflicting results: an increase in risk with number of births (Hildesheim *et al*, 1993) or decrease or no association with parity at all (Lazcano *et al*, 2001). There is still insufficient data to give final conclusions about the effect of number of births on the risk of HPV infections.

In conclusion, our results showed a broad diversity of HPV infections in women with normal cytology, with HR types being at least three times more common than LR types. Whereas young women were particularly at risk of HR and multiple HPV infections, post-menopausal women showed an increased frequency of LR HPV types. In this population, high educational level and multiplicity of sexual partners seem to determine risk among young women. No other hypothesized risk factor was clearly associated with the risk of HPV infection.
